# TDR Thirty Years On: Taking Stock and Envisioning the Future for the Special Programme for Research and Training in Tropical Diseases

**DOI:** 10.1371/journal.pntd.0000314

**Published:** 2008-11-25

**Authors:** Abdallah S. Daar, Susan Reynolds Whyte, Mohamed S. Abdullah, Hu Ching-Li, Stephen L. Hoffman, Martine Berger

**Affiliations:** 1 Departments of Public Health Sciences and of Surgery, University of Toronto, Toronto, Ontario, Canada; 2 Program on Life Sciences Ethics and Policy, McLaughlin-Rotman Centre for Global Health, (University Hospital Network and University of Toronto), Toronto, Ontario, Canada; 3 Institute of Anthropology, University of Copenhagen, Copenhagen, Denmark; 4 Department of Medicine, Aga Khan University Hospital, Nairobi, Kenya; 5 Shanghai Research Center on Care for Children, Shanghai Second Medical University, Shanghai, China; 6 Sanaria Inc., Rockville, Maryland, United States of America; 7 COHRED, Geneva, Switzerland; *PLoS Neglected Tropical Diseases*, United States of America

Between February 2005 and May 2006, we undertook an in-depth, broad-ranging, independent external review of The Special Programme for Research and Training in Tropical Diseases (TDR). TDR, based in Geneva, was created 30 years ago, with the World Health Organization as its executing agency and the World Bank and United Nations Development Programme (UNDP) as initial co-sponsors, with the United Nations Children's Fund (UNICEF) being added in 2003. Its mandate to address research and training in neglected tropical diseases made it a pioneer in its first two decades. But the landscape has changed enormously since its inception, with new sources of funding, different disease patterns, and greater capacity for research and training within disease-endemic countries. Therefore, the Joint Coordinating Board (JCB), TDR's main governing body, requested that this Fourth External Review focus on helping it develop a relevant vision for a future role in this shifting environment. We considered TDR's past and present performance, and its strengths and weaknesses, in order to suggest ways forward.

Because of the nature of TDR's work, especially with its many partners, it is difficult adequately to capture the breadth and depth of its accomplishments in brief. We approached the review as a “case study” [1] guided by the principles of qualitative data collection and analysis. Data were obtained from previous external reviews, internal documents, a management review commissioned by the World Bank, two commissioned papers [2],[3], and other sources. Our main data, however, were from interviews with over 250 people, of whom about 150 were key informants from all major stakeholder groups who participated in face-to-face, in-depth, open-ended interviews, using an interview guide developed for this purpose. Interviewees included TDR staff, including its current director, Dr. Robert Ridley; members of its governing bodies; former directors (Drs. Adetokunbe Lucas [1976–86], Tore Godal [1986–98], and Carlos Morel [1998–2003]; staff of co-sponsor organizations, donor countries, funding agencies, public–private partnerships (PPPs), philanthropies and other global health organizations; countries' representatives; and TDR alumni. We visited various regions to talk to stakeholders and to study institutions, and examined cases illustrative of TDR's work and its relations with others working in global health research and training. Additionally, we made observations of the workings of its various governing bodies and advisory groups, and the Secretariat in Geneva, where our executive secretary was based. We began by asking, Is the original mandate of TDR still valid? Can others discharge this mandate better? What would happen if TDR ceased to exist? If it were to be re-invented for the future, what would the new TDR look like? TDR was given an opportunity to submit comments. These general questions were supplemented by specific ones tailored to particular informants. All were asked to give examples to support their views. The comprehensive material was submitted to a thematic content analysis on the basis of which a taxonomy of issues was developed. Further analyses built on this taxonomy and on continuing discussions and regular “reality checks” with informants. The final 130–page report was completed at the end of May 2006 and is now available on-line, both as a full report [4] and an executive summary [5].

## Findings

In our situational analysis, we examined the double mandate of TDR for research and training. Research capacity strengthening (RCS) remains one of TDR's core distinguishing features,and has been tremendously important for a whole generation of scientists in disease-endemic countries (DECs). However, we found that a gradual shift in TDR's funding has occurred: resources earmarked for research and product development have increased while resources for RCS have stagnated. Efforts were made to mainstream RCS and to include training within product development, but this was not effective. RCS funding was diluted because of the heavy focus on product-oriented capacity building. A renewed commitment to this key function is needed to better coordinate RCS; to leverage new sources of funding and new partners; and to supplement individual training with institutional capacity building tailored to countries' needs. TDR's research mandate is mainly framed in terms of diseases, as its name suggests. In the 30 years since its inception, TDR's portfolio of diseases has grown from five to 10 (see Table 1).

**Table 1:** The Ten Diseases in TDR's Portfolio.

1. Leishmaniasis
2. Schistosomiasis
3. Onchocerciasis
4. Lymphatic filariasis
5. Chagas disease
6. Malaria
7. Leprosy
8. African trypanosomiasis
9. Tuberculosis
10. Dengue

1–8 were the original diseases defined in 1975. The Third External Review was carried out in 1998; TB and dengue were added in 1999. Diagnostics for sexually transmitted diseases were added in 2000. After the Fourth External Review, the Joint Coordinating Board (30th session, 2007) approved TDR's new Ten Year Vision and Strategy encompassing “infectious diseases of poverty”.

We found differing opinions on whether there should be a fixed list of diseases for TDR to address, and if so, which ones. Many of our interviewees suggested that TDR needs to have greater flexibility in this regard and underlined the importance of disease interactions and common control strategies. Moreover, we noted an increasing emphasis on “neglected populations”, not just “neglected diseases”, as the main focus of TDR's future work. Refocusing along these lines will require a broader, more flexible approach to health as a social as well as a medical issue. TDR was a pioneer in supporting trans-disciplinary research [6], but its social sciences staff has remained small. There was only one scientist in the Social, Economic and Behavioural Unit at the time of our review. The ambition to conduct basic research on issues such as social inequality and health sector reform, while also meeting the growing need for intervention research, cannot be realized without substantial strengthening of social science and continued commitment to trans-disciplinary research. Overall, we concluded that TDR has been extremely successful in the past. It worked with industry partners to shape the development of new products, including eflornithine for African trypanosomiasis, praziquantel for schistosomiasis, and various drug combination and formulation innovations for malaria. TDR also sponsored the critical studies establishing the efficacy of insecticide-impregnated bednets for control of malaria. Its more recent successes include the registration of miltefosine for visceral leishmaniasis, facilitation of the sequencing of the *Anopheles gambiae* genome, and provision of evidence for artemisinin-based combination treatments for malaria control policy. Its important social science intervention research includes developing a strategy for managing malaria “close to home”, and contributions in the use of ivermectin for community control of onchocerchiasis in areas with high loiasis. Most importantly, it has played a major role in building individual and institutional research capacity in the developing world (see [7] for a full list of its accomplishments).

Today, TDR continues to be moderately successful, but its influence has waned because of the very changed external landscape. There are many more players and initiatives in neglected diseases research. There are huge new funding sources such as the Bill & Melinda Gates Foundation, the Wellcome Trust, the United States National Institutes of Health, and others; there are PPPs involved in product development [8],[9]; there are other organizations (e.g., Council on Health Research for Development and Global Forum for Health Research) that focus on various research-related issues, including advocacy for health research in developing countries; and there is more research and training conducted in, and by, DECs themselves.

Thus, the future role of TDR at the time of our review was unclear. There was a danger of TDR becoming marginalized as large infusions of funds went elsewhere, for example into product-developing PPPs such as Medicines for Malaria Venture, which had been created with TDR's help. We found that TDR had not managed to partner well with the philanthropies and PPPs or to define respective functions and tasks, or build sufficient linkages and mutual agreements for collaboration. There were difficulties and uncertainties about its relationship with the WHO. It was often over-administered and under-managed, and had sub-optimal in-house communication, inefficient budgetary procedures, inadequate funds, and insufficient discretion to use many of these funds. Some of these problems were caused by forces beyond TDR's control. Much of its staff was demoralized and felt “disempowered”, and its strategic processes needed to be less opaque. It needed to exert more effort in objectively evaluating its work.

Our report documents and discusses the strengths and weaknesses of TDR, summed up in Box 1 (based on [4]). On balance, we found immense support for TDR and widespread conviction that its problems could be addressed and remedied. We concluded that TDR was an extremely valuable organization that could play critical roles not adequately filled by any other organization. Identifying these roles is particularly relevant in the changed external landscape, where new needs are emerging that can only be met by an organization like TDR. Stakeholders were keen to see it develop a strong new vision and to evolve and grow. To do this TDR needed to undergo a process of re-orientation and renewal. It must continue to be supported by all stakeholders with significant increases in funding, and it must have dynamic leadership. The biggest danger was that TDR would resist significant change, believing that it will be the best in the future because it was the best in the past.

**Box 1. TDR's Strengths and Weaknesses**

**Strengths**

- Its nature as a multilateral, inter-governmental agency with multiple co-sponsors.
- Its many competent, caring staff, many of whom are from disease-endemic countries (DECs).
- Its track record (TDR is perceived and emulated as “a star”, “a model”).
- The respect that people have for TDR.
- Its association with WHO, adding to its credibility, especially in DECs, and giving it entry into countries.
- Its record in research capacity strengthening.
- Its values (neutral, science-based, public health orientation, voice of DECs, focused on equity, and access).
- Its many supporters, including its co-sponsors, governing bodies, and alumni.
- Its convening, agenda setting, catalytic and midwifery functions, and leveraging capacity.
- PPPs have in their “pipelines” a number of products—drugs, vaccines, diagnostics—that will need efficient clinical trialing, testing, evaluation, and other forms of intervention research that TDR is well placed to provide.

**Weaknesses**

- Its place in the 21st century among others engaged in “tropical diseases” research and research capacity strengthening is unclear.
- It is embattled by the external environment.
- The voices of DECs could be better represented.
- It is struggling against WHO's and its own bureaucracy.
- It often does not relate well and productively with other entities addressing global health issues.
- It does not sell itself well, especially in articulating its unique strengths.
- It does not make good use of its co-sponsors' resources.
- It has unaddressed management problems.
- In-house creativity is under-emphasized and under-utilized.

## Recommendations

TDR's mandate and institutional base remained largely valid, but needed to be re-interpreted in light of the radically changed external landscape. Evolution and growth must be in both form and function—and form must serve function. Our recommendations for these changes are summarized in Box 2 (based on [4]). We believe that TDR should concentrate on the neglected diseases and health needs of the most needy populations. In the changed landscape, we saw four areas where TDR has a special role to play: 1) Stewardship (including research advocacy and coordination) is a function best undertaken by a neutral international organization, such as TDR, that can maintain an overview of the increasingly complex scene; 2) Expanded intervention research is crucial in order to ensure that new products and strategies actually reach those who need them; 3) Research capacity strengthening for the future is necessary to ensure that DECs have strong research systems and collaborative networks in the South and North; and 4) Research and development for physical products should focus only on those neglected diseases (or areas of neglected diseases) that others (such as the PPPs) are not adequately addressing.

Successful reorientation around these functional areas requires far-reaching institutional transformation. We proposed changes within TDR's Secretariat, in TDR's governance, in its co-sponsor group, in its relationship to WHO, in its way of working with regions and countries, and in its relations to philanthropies, PPPs, the private sector, and other agencies with mandates overlapping TDR's. Only through forging new partnerships will TDR become a flourishing organic part of the new landscape. It must mobilize new resources to evolve. If TDR and its many supporters commit themselves to change, TDR can be re-energized for another 30 years.

**Box 2. Summary of Major Recommendations**

**The 4th External Review Committee recommends that TDR:**

- Is supported by all stakeholders to evolve and grow to a renewed mandate that addresses the very neglected diseases and the health needs of the most needy populations.
- Undertakes a major re-orientation and stakeholder engagement exercise in the very near future. The agenda should include proposals for negotiated/contracted strategic alliances and partnerships with major stakeholders; proposals to secure increased funding; discussions with co-sponsors on how best to make use of the latter's resources; a mechanism for regular consultations to coordinate approaches and investments.
- Creates four functional areas as follows:
  1. *Stewardship*, *Research Advocacy*, *and Coordination:* a role that no other institution at present can legitimately fulfill; requires a cultural change in TDR.
  2. *Expanded Intervention Research:* rapidly scale up; include all neglected diseases and health needs of the most needy; play a key role in the evaluation of new products from Phase I clinical trials through Phase IV post-marketing studies; study the effectiveness of intervention strategies in real-life situations; emphasize policy and social, economic, and behavioural research; clarify and reinforce links with WHO's control programmes.
  3. *Research Capacity Strengthening for the Future:* foster not only technical skills but also competences in research oversight and management, as well as ethics; reinvigorate efforts to strengthen and collaborate with research training institutions in the South and build more effectively on the RCS potential of its networks of alumni, and of scientists in the diaspora.
  4. *Research and Development for Physical Products:* reduce R&D for physical products to address only the few very neglected diseases (or areas of neglected diseases) that others are not addressing adequately.
- Decentralizes minimally: create small, region-based TDR Teams to increase alignment with countries' needs and priorities, ownership, sustainability, and ability to draw on the local resources of all TDR co-sponsors and alignment with their country focus.
- Develops a strategic staffing plan: take into account the needs of the new TDR and its future functions and structure.
- Considers enlarging the co-sponsor group: reflect the key players in global health and the new sources of major funding for global health research, RCS, and public health interventions.
- Considers ways to improve its relations with PPPs, the private sector, philanthropies, and others who have similar or overlapping mandates.
- Improves its relationship with WHO, its executing agency: clarify unresolved issues in the Memorandum of Understanding and negotiate a comprehensive Administrative Structural Agreement to make its administrative and financial management more efficient and transparent, and to enable changes needed for renewal and implementation of its new directions.
- Improves its governance mechanisms: clarify the relationships between its components and allow for bigger support roles e.g., advocacy and resource mobilization by the Joint Coordinating Board, increasing the latter's engagement with the Programme and fully reclaiming its governance role, with definitive authority on priority-setting and an active role in policy-making.
- Has strong leadership: the next director should be given greater authority, independence, and seniority of decision-making (with corresponding higher salary level); leadership qualities include being a good scientist; being a decisive, nimble, bold, and courageous visionary; possessing strong diplomatic and political skills; being a great communicator who is internationally respected; being able to take responsibility for major decisions; being comfortable working with all stakeholders; being able to live and work in disease-endemic countries; and being able ultimately to manage the whole TDR Secretariat and overrule petty bureaucracy.
